# Synergy between TMZ and individualized multimodal immunotherapy to improve overall survival of IDH1 wild-type MGMT promoter-unmethylated GBM patients

**DOI:** 10.1038/s41435-022-00162-y

**Published:** 2022-02-16

**Authors:** Stefaan W. Van Gool, Jennifer Makalowski, Michael Bitar, Peter Van de Vliet, Volker Schirrmacher, Wilfried Stuecker

**Affiliations:** Immun-Onkologisches Zentrum Köln (IOZK), Hohenstaufenring 30-32, 50674 Köln, Germany

**Keywords:** Translational immunology, Immunization, Tumour immunology

## Abstract

The prognosis of IDH1 wild-type MGMT promoter-unmethylated GBM patients remains poor. Addition of Temozolomide (TMZ) to first-line local treatment shifted the median overall survival (OS) from 11.8 to 12.6 months. We retrospectively analyzed the value of individualized multimodal immunotherapy (IMI) to improve OS in these patients. All adults meeting the criteria and treated 06/2015–06/2021 were selected. Thirty-two patients (12f, 20m) had a median age of 47 y (range 18–69) and a KPI of 70 (50–100). Extent of resection was complete (11), <complete (12) or not documented (9). Seven patients were treated with surgery/radio(chemo)therapy and subsequent IMI (Group-1); 25 patients were treated with radiochemotherapy followed by maintenance TMZ plus IMI during and after TMZ (Group-2). Age, KPI and extent of resection were not different amongst both groups. The median OS of group-1 patients was 11 m (2 y OS: 0%). Surprisingly the median OS of group-2 patients was 22 m with 2 y OS of 36% (CI95%: 16–57), which was significantly (Log-rank: *p* = 0.0001) different from group-1. The data suggest that addition of IMI after local therapy on its own has no relevant effect on OS in these GBM patients, similar to maintenance TMZ. However, the combination of both TMZ + IMI significantly improved OS.

Glioblastoma multiforme (GBM) is an orphan cancer disease, causing the highest number of years of life lost due to cancer in human [1]. Standard of care in 2021 consists of maximal safe surgery followed by radiochemotherapy and maintenance chemotherapy with Temozomide (TMZm). The pivotal randomized clinical trial (RCT) to prove efficacy of TMZ was published in 2005 and 2009 [2]. Increasing research exists in the domain of anti-angiogenesis, targeted therapy, physics therapy like tumor-treating fields, biological treatments with oncolytic viruses or different kinds of immunotherapy. One highly dominating factor for prognosis, now in some clinical trials used as in/exclusion criteria or for stratification, is the methylation status of the MGMT promoter [3]. Besides, other factors play a role in the ultimate outcome of the patient like the clinical status of the patient, reflected in the Recursive Partitioning Analysis [2], and molecular characteristics of the GBM, summarized as GBM-molRPA classification [4]. When treatments focus in part on the tumor-host interaction and the patient’s immune system, other factors like the ImmunoScore of the tumor, the presence of different types of myeloid cells and microglia, and the systemic immune system in all its functional compartments play a role as well [5]. Besides the diversity of all these prognostic factors, the dynamic change of subclones within the tumor, the changing tumor-host interaction and the changing immune functioning upon other anticancer and supportive treatments make the design of RCTs with pre-defined study and control patients, treated within fixed protocols, challenging [5]. Therefore, new, even preliminary observations in the domain of innovative personalized medicine keep their value to gain novel insights and generate new hypotheses.

The Immune-oncologic centre Köln (www.IOZK.de) has the approval (DE_NW_04_MIA_2015_0033 and DE-NW-04-MIA-2020-0017) to produce IO-Vac^®^ since May 2015: monocyte-derived autologous mature dendritic cells (DCs) loaded with autologous tumor antigens and matured in the presence of IL-6, TNF-α, and IL-1β in combination with Newcastle Disease Virus (NDV) as danger signal. The treatment concept of Individualized Multimodal Immunotherapy (IMI) consists of 1/immunogenic cell death (ICD) [6] therapy with repetitive bolus injections of NDV and sessions of local modulated electrohyperthermia (www.Oncotherm.com), 2/active specific immunotherapy: vaccination with IO-Vac^®^, 3/modulatory immunotherapy, personalized for each patient over time, and 4/complementary medicines mostly upon initiative of the patient. Treatment details and clinical experiences have been reported [7].

The role of ICD induction by NDV, thereby changing the tumor microenvironment and eliciting an anti-GBM immune protection, was demonstrated in the GL261 preclinical mouse model [8]. Modulated electrohyperthermia is known to induce ICD as demonstrated in preclinical models [9] and in clinical setting [10]. The strong antigenicity of ICD-killed tumor cells for loading DCs resulted in a vaccine with superior immunogenicity [11]. The role of DC vaccination during the first-line standard of care for GBM patients has been studied world-wide in several studies, amongst the HGG-2010 study (EudraCT 2009-018228-14) [12, 13]. DC vaccination in this RCT was scheduled after radiochemotherapy, and boost vaccines with lysate were given during TMZm. In the control arm, similar immunotherapy was scheduled after TMZm. Data suggested the 2-year overall survival (OS) being higher in the control arm [14]. Because of this, we introduced since June 2015 a novel concept to strengthen the tumor control during maintenance chemotherapy with ICD therapy given from day 8 to 12 after each 5-day TMZm treatment. Only after all chemotherapy and expected maximal tumor reduction, patients received active specific immunotherapy with two IO-Vac^®^ treatments to install an anti-GBM immune protection. DCs were loaded with ICD-induced serum-derived antigenic extracellular microvesicles and apoptotic bodies and tumor lysate when available. Finally maintenance ICD treatments (ICDm) were recommended to include potentially developing novel tumor subclones within the installed immune protection [5, 7, 15]. All patients were treated in compassionate use (“individueller Heilversuch”).

Current data are derived from a new retrospective analysis of treated patients between 27 May 2015 and 08 June 2021. Records from 5576 patients were present in the database; 742 patients started treatment during the mentioned time period; 308 of them had brain tumor; 194 patients had GBM; 97 patients were treated with IMI as part of first-line combined treatment, 74 of them had age between 18 and 75 years. Patients indicated in the database as IDH1 mutation, low grade astrocytoma disease history, diffuse midline glioma genetics, or history of second malignancy GBM were excluded. Patients, who all had slightly different individualized treatment schedules, were arbitrary categorized in three groups: 1/IMI after local treatment (surgery with our without radio(chemo)therapy, but without TMZm + ICD therapy); 2/ICD treatment during TMZm (at least one cycle) followed by DC vaccination and maintenance ICD therapy; 3/patients starting IMI after completion of standard of care. The decision when and how IMI was inserted into the first-line treatment as “individueller Heilversuch” was taken individually by each patient after an immune evaluation, an extensive informed consent and an immune-diagnostic procedure. Sixty-six patients belonged to group-1 and group-2, 32 of them being noticed in the database as MGMT promoter-unmethylated.

Seven patients (5 females, 2 males) were categorized as group-1, 25 patients (7 females, 18 males) as group-2. There were no differences in age (median 47.5 y, ranging 18–69y), in Karnofsky performance index (median 85 ranging 50–100), or extent of resection (complete:11, less than complete: 12, not documented: 9) between both groups (Table 1). Immune diagnostic blood variables were categorized as below (L), within (N) or above (H) the normal range for each variable. We found no proportional differences in the two treatment groups. The percentage of patients without circulating tumor cells (CCC, https://www.biofocus.de/molecular-oncology), with CCC but negative for mRNA expression for PDL1, and with CCC but positive for mRNA expression for PDL1 was also equal in both groups.

**Table 1:** Patient data.

	Group-1			Group-2		
Clinical data						
	P25	Median	P75	P25	Median	P75
Age	36	49	69	41.5	46	57.5
KPI	50	70	95	70	90	100
	**R0**^**a**^	**R1**^**b**^	**ND**^**c**^	**R0**	**R1**	**ND**
Surgery	1	4	2	10	8	7
**Laboratory data**						
	**L**^**d**^	**N**^**e**^	**H**^**f**^	**L**	**N**	**H**
Hemoglobin		7		3	21	1
White Blood cells	1	5	1	2	19	4
Platelets	4	3		5	20	
T cells	2	4		13	12	
B cells	6			22	2	1
NK cells	3	3		15	10	
NK cell function	5		1	13	8	2
CD4 IFNg	1	4		1	19	2
CD4 IL4	1	5		2	13	8
	**CCC**−^**g**^	**CCC** **+** **PDL1−**^**h**^	**CCC** **+** **PDL1+**^**i**^	**CCC−**	**CCC** **+** **PDL1−**	**CCC** **+** **PDL1+**
CCC	2	2	1	9	9	5
**Treatment data**						
***ICD therapy***						
Total NDV^j^ injections	6	15	24	24	42	47
Total mEHT^k^ sessions	4	11	24	17	39	46
***Active specific immunotherapy with IO-Vac***^***®***^						
	**P25**	**Median**	**P75**	**P25**	**Median**	**P75**
IO-Vac^®^	1	2	2	1	2	2
Total DCs (×10^6^)	11.6	15.4	38.3	7.2	24	36.45
***Source of tumor antigens***						
	**mEV**^**l**^	**TL**^**m**^	**mEV** **+** **TL**	**mEV**	**TL**	**mEV** **+** **TL**
	6	1		14	1	5

^a^Documented total resection.

^b^Documented less than total resection.

^c^Extent of resection not documented.

^d^L = lower than laboratory reference level.

^e^N = within laboratory reference level.

^f^H = above laboratory reference level.

^g^CCC−: no circulating cancer cells detected in blood (www.biofocus.de).

^h^CCC + PDL−: CCC detected, but mRNA expression for PDL1 below limit.

^i^CCC + PDL1+ : CCC detected, and mRNA expression for PDL1 above limit.

^j^NDV: Newcastle Disease Virus.

^k^mEHT: modulated electrohyperthermia.

^l^DCs loaded with ICD therapy-induced serum-derived antigenic extracellular microvesicles and apopotitic bodies.

^m^DCs loaded with tumor lysate.

Both treatment groups received a similar amount of IO-Vac^®^ vaccines (median 2, ranging 0–4), and an equal total number of DCs (median 23 × 10e6, ranging 0–104,8 × 10e6). Source of antigen were ICD therapy-induced antigenic extracellular microvesicles and apoptotic bodies eventually with tumor lysate (*n* = 7). Patients from group-2 received significantly more ICD therapies as compared to group-1 (Table 1). This was due to the ICD therapy sessions during the TMZm, but also due to longer survival and hence more maintenance ICD therapies after the IO-Vac^®^ vaccines.

Because MRIs were not centrally reviewed, we did not focus on progression-free survival, which is difficult to assess [16], but only OS in this retrospective analysis (Fig. 1). The median OS of group-1 patients was 11 m (2 y OS: 0%). One patient of group-2 was lost of follow up. Surprisingly the median OS of the other 24 group-2 patients was 22 m with 2 y OS of 36% (CI95%: 16–57), which was significantly (Log-rank: *p* = 0.0001) different from group-1. The prolonged OS in group-2 illustrates former data from meta-analyses that the addition of active specific immunotherapy significantly prolongs OS of GBM patients [17–21]. At time of database fixation, 9 of 24 patients were alive with a median follow up of 20.3 months (ranging 8.7–32.1). Three of them were still in first-line treatment plan 32, 28 and 8 months after diagnosis. Similar to literature findings, we did not detect IMI-related major toxicities registered in the database [21]. Because patients were treated in compassionate use outside a clinical trial, and because there was lack of knowledge of tumor-specific neoantigens (except for one patient [5]), immune-monitoring tests were not performed.

**Fig. 1:**
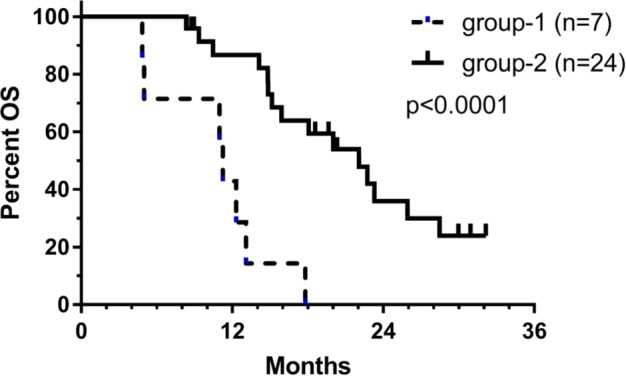
Overall survival of both patient groups. Group-1 patients (7 patients) received individualized multimodal immunotherapy after local therapy (surgery and/or radio(chemo)therapy). Group-2 patients (25 patients, one loss of follow-up) received ICD therapy during TMZ cycles (at least one cycle) followed by active specific immunotherapy with dendritic cell vaccines (IO-Vac^®^) after all chemotherapy.

The IMI strategy is based on insights gained from basic science and translational medicine research [15]. Neurosurgery, radiotherapy, and chemotherapy are the standard anti-cancer treatment pillars. Two novel anti-cancer treatment pillars are emerging: 1/targeted (immuno)therapies (CAR T cells belong to this pillar) based on the molecular structure of the tumor cells, and 2/physics and biologic therapies, exemplified by electromagnetic waves, respectively several oncolytic viruses. Although all treatments are aimed in the first place to kill tumor cells, ICD induction is more or less included in their spectrum of activities thereby generating an immune response [6, 22]. In particular, the working mechanism of modulated electrohyperthermia and oncolytic virus therapy is to a large extent based on the induction of ICD and generation of an immune protection. One patient has been reported who did not show a tumor-specific anti-GBM immune response after radiochemotherapy and 5 cycles of maintenance temozolomide (TMZ), but a clear CD4 and CD8 response after receiving 7 ICD therapy courses along the 7 further maintenance TMZ cycles, plus one subsequent DC vaccine in which the immature DCs were loaded with ICD therapy-induced serum-derived antigenic extracellular microvesicles and apoptotic bodies [5]. Active specific immunization with DCs should be installed after the maximal first-line anticancer treatment. The aim is then to install a broad long-term immune protection against the different known and unknown tumor antigens. This protection should be maintained and further broadened with subsequent ICD maintenance treatments. Finally, modulatory immunotherapies like checkpoint inhibitors, therapies against regulatory T cells or myeloid-derived suppressor cells, and therapies against pro-tumoral inflammation should be added to maximize the immunization and sustain the immune protection. Such combined and personalized treatment approach should take into account the dynamics of all biologic processes in the patient [5].

The finding of a synergy between TMZ and IMI to improve OS for IDH1 wild-type MGMT promoter-unmethylated GBM patients is remarkable in the light of the negative repercussions of TMZ on immune cells [23]. The effect of TMZ in the context of the tumor-host interaction is complex. TMZ functions as a direct anti-cancer alkylating drug [24] and during radiochemotherapy as a radiosensitizer [25]. After radiochemotherapy, the systemic lymphocytic pool is depleted. However, detailed analysis of the immune function during maintenance TMZ chemotherapy showed already recovery of the proliferative capacity of T cells [23], which is the time when patients from group-2 started ICD therapy. Moreover, TMZ might reduce PDL1 expression on tumor cells [26] making them more prone for anticancer effector cytotoxic T cells. TMZ might reduce the load of regulatory T cells in the tumor microenvironment [27]. The TMZ-induced mutagenesis [28, 29] might contribute to new tumor subclones with changed antigenicity. In the presented IMI strategy the changing antigenicity is picked up by our IO-Vac^®^ vaccines scheduled at the end of the maintenance TMZ. We indeed mostly did not use the antigens of the original tumor tissue, and included always ICD-induced serum-derived antigenic extracellular microvesicles and apoptotic bodies yielded at time of vaccination and reflecting the actual antigenic profile in the mutated residual tumor cells after chemotherapy.

The authors are aware that these hypothesis-generating data, presented as Brief Communication, are based on retrospectively analyzed findings in a small group of patients. Nevertheless, whereas IMI after only local therapy (surgery without or with radio(chemo)therapy) did not improve the median OS, and the addition of TMZ during and after radiotherapy only marginally (albeit significantly) improved median OS from 11.8 to 12.6 months [2], the major shift in median OS from 11.8 to 22.07 months when both TMZ and IMI were combined together suggests a synergy between both modes of action for the improvement of the OS of patients with MGMT promoter-unmethylated GBM patients.
